# Risk of Falls in Parkinson's Disease: A Cross-Sectional Study of 160 Patients

**DOI:** 10.1155/2012/362572

**Published:** 2012-01-15

**Authors:** Ana Contreras, Francisco Grandas

**Affiliations:** ^1^Unidad de Investigación en Trastornos del Movimiento, Instituto de Investigación Sanitaria Hospital Gregorio Marañón, 28007 Madrid, Spain; ^2^Servicio de Neurología, Hospital General Universitario Gregorio Marañón, C/ Doctor Esquerdo 46, 28007 Madrid, Spain

## Abstract

Falls are a major source of disability in Parkinson's disease. Risk factors for falling in Parkinson's disease remain unclear. To determine the relevant risk factors for falling in Parkinson's disease, we screened 160 consecutive patients with Parkinson's disease for falls and assessed 40 variables. A comparison between fallers and nonfallers was performed using statistical univariate analyses, followed by bivariate and multivariate logistic regression, receiver-operating characteristics analysis, and Kaplan-Meier curves. 38.8% of patients experienced falls since the onset of Parkinson's disease (recurrent in 67%). Tinetti Balance score and Hoehn and Yahr staging were the best independent variables associated with falls. The Tinetti Balance test predicted falls with 71% sensitivity and 79% specificity and Hoehn and Yahr staging with 77% sensitivity and 71% specificity. The risk of falls increased exponentially with age, especially from 70 years onward. Patients aged >70 years at the onset of Parkinson's disease experienced falls significantly earlier than younger patients.

## 1. Introduction

Falls are a major source of morbidity and disability in Parkinson's disease (PD). The risk of falls is increased in patients with PD [1], and the findings of several studies have revealed that 38 to 87% of parkinsonian patients experienced falls [2–5]. Falls are commonly a recurrent phenomenon in PD. One meta-analysis of several prospective studies showed that the rate of recurrent falling over a three-month period was 57% among those patients who had reported previous falls [6].

Direct consequences of falling are fractures, particularly hip fractures, head trauma, contusions and other injuries [5, 7–9], and even death [10]. In addition, falling may induce fear of new falls [11], which can in turn reduce mobility and lead to osteoporosis, loss of independence, social isolation, and depression [12]. Moreover, falls increase the risk of admission of PD patients to hospitals [13] and nursing homes [5]. The economic burden of falls in PD is very high and it is estimated that the direct medical costs of PD fallers double those of nonfallers [14].

Preventing falls has become one of the most important unmet needs in PD, and potential strategies to prevent falls should focus on patients at higher risk for falling. Therefore, identifying risk factors is of paramount importance.

Studies aimed at finding such risk factors have been inconclusive to date. Retrospective studies have brought out inconsistent results. Thus, falls in PD have been related to age [15], disease duration [16, 17], disease severity [3, 17], autonomic dysfunction [15], urinary incontinence [4], increased time in the get-up-and-go test [4], greater postural sway [18, 19], poorer stability in response to pushes and pulls [20], and variability of stride time [17]. Other proposed predictors of falls include poor standing balance [21, 22], dyskinesia, dementia, frontal impairment, freezing of gait [23], orthostatic hypotension, and muscle weakness [24].

The best predictor of falling in PD found in a meta-analysis of prospective studies with follow-up periods of three months [2, 3], six months [25], and twelve months [26] was suffering two or more falls in the previous year [6]. Although this conclusion reinforces the concept of recurrent falls in PD, it does not help to identify PD patients at risk before the first fall. A more recent prospective study with a six-month follow-up in patients with early-stage PD failed to identify risk factors for the first fall apart from increased postural sway when standing on a firm and foam surface with the eyes open in the group of fallers [27]. The follow-up period of these studies might have been insufficient to assess the prospective predictiveness of aging or disease progression on the appearance of falls, if these variables were relevant risk factors for falling in PD.

To try to elucidate the relevant risk factors for falling in PD, we carried out a cross-sectional study of a group of unselected patients of different ages and disease duration, taking into account most of the clinical variables potentially associated with falls.

## 2. Methods

### 2.1. Subjects

The study sample comprised consecutive patients with PD who attended the Movement Disorders Clinic of Hospital Universitario Gregorio Marañón during a nine-month period. These patients were regularly followed up with visits to the clinic every 3-4 months. The diagnosis of PD was confirmed according to the United Kingdom Parkinson's Disease Brain Bank criteria [28]. Patients who underwent functional stereotactic surgery for PD were excluded. The local ethics committee approved the study and all participants gave their informed consent. All patients were interviewed and examined by the authors.

### 2.2. Assessment of Falls

A fall was defined as an event which resulted in the patient unintentionally coming to the ground or other lower level not as a result of a major intrinsic event or overwhelming hazard [29, 30]. The patients were questioned about the existence of these events since the onset of PD. Information about the time of the first fall (year, month) was obtained from the patients and checked with relatives, caregivers, and clinical records for accuracy of data. In cases with more than one fall, the number of falls in the last year, and particularly in the last day, week, month, three-month period, and six-month period, was recorded. Fall-related injuries, especially fractures, were also recorded.

### 2.3. Variables

We recorded gender, age, age at onset of PD, initial predominant symptom (tremor or akinetic-rigid syndrome), disease duration, and the presence of motor fluctuations and dyskinesia.

We also recorded treatments with antiparkinsonian drugs including L-dopa (L-dopa/carbidopa, L-dopa/benserazide, controlled-release L-dopa formulations), COMT inhibitors (entacapone, tolcapone), MAO-B inhibitors (selegiline, rasagiline), amantadine, anticholinergics, and dopamine agonists (bromocriptine, pergolide, cabergoline, pramipexole, ropinirole, transdermal rotigotine, subcutaneous apomorphine) until the date of the survey (nonfallers) or the date of the first fall. Only sustained treatment for more than two months at recommended doses was considered. Treatment with benzodiazepines, antidepressants, atypical neuroleptics (quetiapine, clozapine), and cholinesterase inhibitors was also recorded in the same manner.

History of syncope and symptoms of orthostatic hypotension (light-headedness, dizziness, weakness on standing from sitting or lying position) [31] were recorded as were past history of stroke, hypertension, and diabetes.

Clinical data were obtained from the patients and checked with relatives, caregivers, and clinical records for accuracy.

All patients underwent neuroimaging studies (cranial-computed tomography, brain magnetic resonance imaging [MRI], or both), which were reviewed by a neuroradiologist to identify cases with moderate/severe leukoaraiosis and infarcts of any size and location. All PD fallers underwent brain MRI after the onset of falls.

The motor function subscale (part III) of the Unified Parkinson's Disease Rating Scale (UPDRS) [32] was administered, as were the Schwab and England activities of daily living [33], Hoehn and Yahr staging [34], Mini Mental State Examination [35], Tinetti's Gait and Balance functional test [36] (the appendix), and the freezing of gait questionnaire [37].

The timed get-up-and-go test [38] was performed and a ten-meter walk at the preferred speed was timed, videotaped, and used to calculate gait velocity, and step length and cadence.

In fluctuating patients, UPDRS, Schawb-England, and Hoehn and Yahr scales and timed tests were administered in off situation (8–10 hours after patients stopped their usual antiparkinsonian treatment) to evaluate the possible influence of disease severity.

### 2.4. Statistical Analysis

Mean differences between fallers and nonfallers were assessed using the *t*-test for independent samples or the Mann-Withney *U* test for continuous variables with parametric or nonparametric distribution, respectively. The *χ* ^2^ test was used to assess associations between categorical variables.

Variables with statistically significant differences between fallers and nonfallers were entered into bivariate and stepwise multivariate logistic regression analyses with the dichotomous criterion of falls as the common regressor to determine the best explanatory independent variables. Several stepwise multivariate logistic regression models were tested, evaluating all possible combinations of the variables. Up to five variables were considered in each model.

Receiver operating characteristic (ROC) analyses were performed to assess the sensitivity and specificity of each variable in predicting fallers. The point that simultaneously maximized sensitivity and specificity was selected as the cut-off value. Accuracy was calculated based on the proportion of correctly classified cases using cut-off values.

Kaplan-Meier curves were used to evaluate the relationship between time to onset of falls and age and disease duration. The log-rank test stratified by age at onset of PD was performed to evaluate the effect of age at onset on the appearance of falls during the course of the disease. A *P* value <0.05 was considered significant in all tests.

Data were analysed using the Statistical Package for the Social Sciences (SPSS) version 15.0 for Windows.

## 3. Results

The study sample comprised 160 patients with PD (72 men, 88 women, mean age 72 ± 9.5 years). Demographic and disease characteristics are described in Table 1. Sixty-two patients (38.8%) reported at least one fall since the onset of PD, and 42 of these patients were recurrent fallers (68% of patients with falls). The average frequency of falls in the previous year in recurrent fallers was as follows: one or more falls per day, 4.8%; one fall per week, 9.7%; one fall per month, 25.8%; one fall every six months, 59.7%.

Falls led to fractures in 20 patients (32.2% of fallers) and to bruises, skin lacerations, and other injuries in a further 16 cases (25.8% of fallers).

At the first fall, mean age was 70.7 ± 9.6 years and the mean disease duration when the first fall occurred was 7.2 ± 6 years.

Thirty-two patients were unable to perform timed tests because they could not walk unaided (Hoehn and Yahr stages IV and V).

### 3.1. Comparison between Fallers and Nonfallers

Fallers were older and had longer disease duration and increased disease severity according to the UPDRS (part III), Hoehn and Yahr and Schwab and England activities of daily living scores. In addition, fallers scored worse in the Mini-Mental State Examination and experienced a higher frequency of motor fluctuations, dyskinesia, and freezing of gait (Table 1). Tremor as the initial predominant symptom was more frequent in nonfallers. Fallers were treated with higher doses of levodopa and more frequently received COMT inhibitors, central cholinesterase inhibitors, and atypical neuroleptics (quetiapine) than nonfallers (Table 2). Patients treated with central cholinesterase inhibitors (rivastigmine) had dementia associated with PD and those treated with quetiapine had hallucinations.

No differences were observed between fallers and nonfallers in other drug treatments, age at onset of PD, symptoms of orthostatic hypotension and cerebrovascular disease (clinical, neuroimaging, and risk factors).

Fallers scored worse in the Balance and Gait subscales of the Tinetti functional test and were slower in the timed get-up-and-go test (Table 2). There were no statistically significant differences in gait velocity, step length, and cadence between fallers and nonfallers.

### 3.2. Regression Analysis

The independent variables identified as significantly associated with falls in the bivariate logistic regression were the same as those that had been found to be statistically different in the previous approach, except for the timed get-up-and-go test, which lost its statistical significance (Table 3).

When these variables were included in stepwise multivariate models of logistic regression, only the Tinetti Balance functional test was independently associated with falls (OR = 0.847, 95%  CI = 0.740–0.971, *P* = 0.017). The rest of the variables lost their statistical significance once Tinetti Balance subscale entered into the regression model. In Table 3 appear the most favorable OR obtained for the other variables, which did not reach in any case the threshold of statistical significance.

### 3.3. ROC Analysis

**Figure 1:**
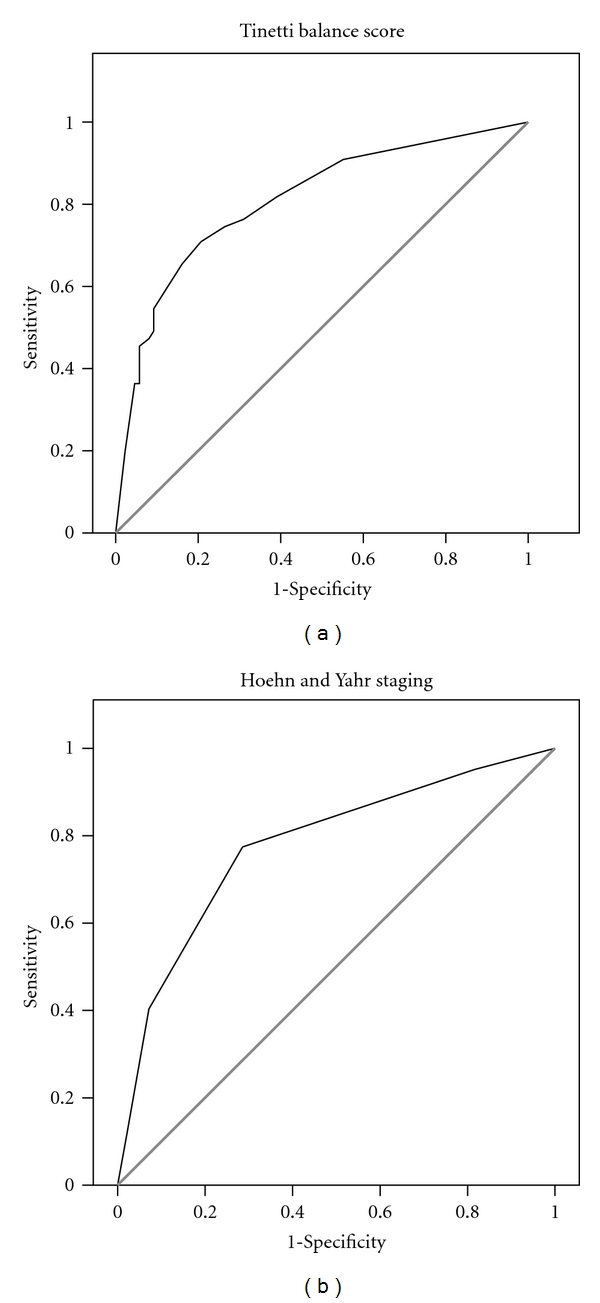
Receiver operating characteristic curves of the Tinetti Balance score (Figure 1(a)) and Hoehn and Yahr staging (Figure 1(b)). AUC, sensitivity, specificity, and accuracy are shown in Table 4.

Table 4 shows the outcomes of the ROC analysis for the nondichotomous variables associated with falls in the bivariate logistic regression. Again the Tinetti Balance functional test showed the highest combination of sensitivity and specificity (71% and 79% resp.) for predicting falls followed by Hoehn and Yahr staging (77% and 71%), with an accuracy of 76% and 74%, respectively, (Figures 1(a) and 1(b)). The combination of these two variables increased specificity to 80% and accuracy to 77%. Seventy-seven per cent of fallers were in Hoehn and Yahr stage ≥3 whereas 72% of nonfallers were in stages 1 and 2 (Table 1).

### 3.4. Kaplan-Meier Curves

**Figure 2:**
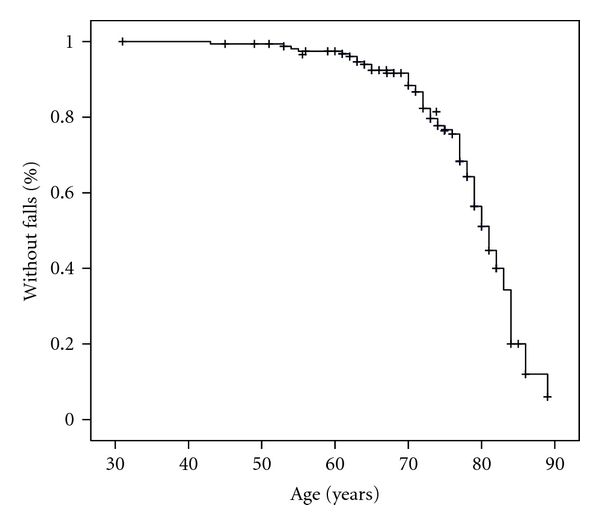
Kaplan-Meier curve showing that the risk of falls increases exponentially with age, mainly from the age 70 onward.

**Figure 3:**
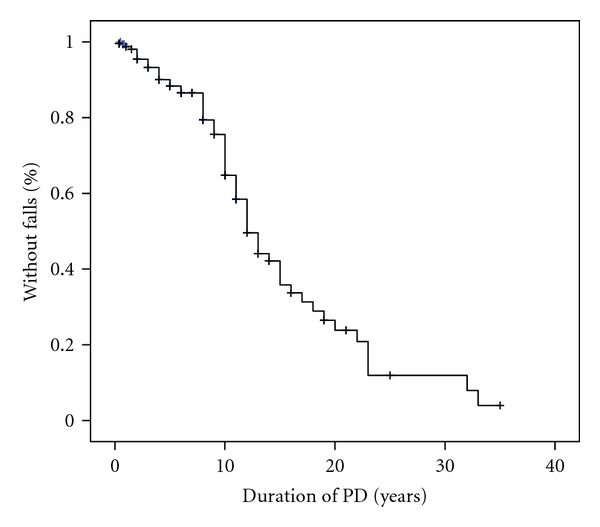
Kaplan-Meier curve showing that the risk of falls increases with the duration of Parkinson's disease.

**Figure 4:**
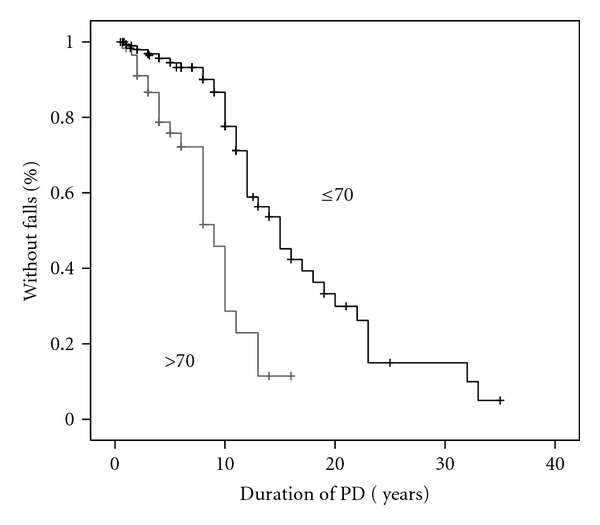
Kaplan-Meier curve showing the effect of age at onset of Parkinson's disease on the appearance of falls. Patients with age at onset >70 years fall earlier than younger patients (log rank *P* < 0.001).

Survival curves show that the risk of falls increased exponentially with age, particularly from the age of 70 years (Figure 2). In addition, the prevalence of falls increased with the duration of PD (Figure 3). The combined effect of age at onset of PD and disease duration is illustrated in Figure 4. Patients who developed PD after the age of 70 years experienced falls significantly earlier than younger patients (log rank, *P* < 0.001).

## 4. Discussion

The first fall is a milestone in the life of patients with PD, and it is usually recalled with reasonable accuracy by patients and relatives. In the present survey, the prevalence and frequency of falls and the morbidity they caused were similar to those of other reported series [5, 39]. In short, we confirmed that falls are frequent and recurrent in patients with PD and responsible for fractures in about one-third of fallers and for other relevant injuries in a further 25% of patients.

Using a series of statistical methods, we found that the independent variables most associated with falls were the Tinetti Balance score and Hoehn and Yahr staging. The Tinetti Balance test predicted falls in our patients with 71% sensitivity and 79% specificity, and Hoehn and Yahr staging predicted falls with 77% sensitivity and 71% specificity.

The Tinetti test is a simple, widely used, qualitative test comprising two subscales, one to assess clinical balance and another to assess gait [36]. The balance subscale consists of nine items, where lower scores indicate poor balance. The Tinetti test is a reliable and valid clinical test to measure balance and gait in elderly people and in patients with PD [40]. We found the Tinetti Balance subscale to be a useful tool for assessing the risk of falls in PD with even higher accuracy than the total Tinetti test score.

In our study, most PD fallers were Hoehn and Yahr stage III or more. Thus, the transition from stage II to III, with the emergence of postural instability, plays a crucial role in the appearance of falls and is related to increased disability in many gait-dependent activities [41]. As expected, balance dysfunction seems to be the main cause of falls in patients with PD. Postural instability in PD is caused by deficits in several components of postural control, such as hypometric preparatory adjustments, delayed reaction time, abnormal automatic postural reactions, and abnormal axial kinesthesia [39, 42, 43]. Postural instability occurs in the course of PD as a consequence of disease progression. Therefore, fallers had longer disease duration and increased disease severity based on the UPDRS, Hoehn and Yahr and Schwab-England activities of daily living scores, and more frequently experienced motor fluctuations and dyskinesia. For the same reasons, fallers were treated with higher doses of levodopa and more frequently used COMT inhibitors. These results are in keeping with similar findings from other studies [3, 5, 17, 25].

Freezing of gait was more frequent among fallers, although its statistical relevance was overcome by postural instability. Freezing of gait can precipitate falls in unstable patients [12] and could be the principal cause of falls in a subgroup of patients with PD.

Tremor as the initial predominant motor symptom was more frequent among nonfallers. This finding is consistent with the slower clinical progression to Hoehn and Yahr stages III to V found in parkinsonian patients with a tremor-dominant clinical subtype in prospective clinicopathological studies [44].

MMSE scores were worse in fallers. This may reflect the interaction between cognitive function and gait and posture abnormalities [45, 46], although executive function and attention were not specifically assessed in our study. On the other hand, cognitive decline might be an epiphenomenon related to more advanced disease in PD fallers.

In our series, there was no statistical significant difference in history of symptoms of orthostatic hypotension between fallers and nonfallers, although blood pressure measurements in supine and standing positions were not performed.

An interesting finding was that the risk of falls in PD increased exponentially with age, especially from 70 years onward. Thus, ageing seems to play an important role in advanced PD, perhaps by hastening the underlying disease process, thus allowing neuropathological changes to spread rapidly to neural structures related to gait and balance control in the late stages of the disease [47]. Furthermore, in our survey, patients who developed clinical symptoms of PD after the age of 70 experienced falls significantly earlier than those with a younger onset, illustrating the combined effect of ageing and disease progression. In fact, age at onset of PD should be taken into account when considering early falls as a red flag that may question the clinical diagnosis of PD.

A limitation of this study is the retrospective assessment of falls. It has been suggested that elderly people tend to forget previous falls [48]. However, the accuracy of dating past falls may depend on how subjects are interviewed. We managed to date falls, particularly the first one, with the combined information of patients, relatives, caregivers, and clinical records. In addition, the survival curve for time to the first fall after the onset of PD in our analysis was similar to that of the only long-term study of patients with PD in which falls were prospectively assessed [5], although it was not designed to investigate risk factors of falling. This implies that our survey, despite its cross-sectional design, does not present important biases in the assessment of falls.

Although the predictive value for falls in PD of the Tinetti test and Hoehn and Yahr staging is limited, and other more refined tests of balance deficits in PD should be developed, the use of these simple and rapid clinical tests may help identify high-risk patients.

In elderly people without PD, exercise programs specifically targeting balance have proven to be particularly effective in preventing falls [49]. Targeted exercise improves balance in PD [50], and cueing training can improve freezing of gait [51]. In addition, a recent study has suggested that the treatment with central cholinesterase inhibitors may reduce falls in nondemented PD patients [52].

Research efforts should be directed toward finding better predictors of falls in PD— perhaps using posturography [53] or other electrophysiological devices for testing postural stability [54]—and toward developing therapeutic strategies to improve balance and prevent falls in these patients.

## Figures and Tables

**Table 1:** Demographic and disease characteristics^a^.

	All patients (*n* = 160)	Nonfallers (*n* = 98)	Fallers (*n* = 62)	Test^b^	*P* value
Age (*y*)	72.0 (9.5)	70.6 (9.6)	74.2 (8.9)	1	0.012
Male (%)	52.5	47.9	53.2	2	0.884
Age at onset (*y*)	63.9 (11,2)	64.2 (11,1)	63.5 (11.3)	1	0.682
Disease duration (*y*)	8.1 (6.4)	6.4 (5.4)	10.6 (7.0)	3	<0.001
PD subtype at onset				2	0.012
TDT (*n*, %)	100 (62.5)	69 (70.5)	31 (50.0)		
ART (*n*, %)	60 (37.5)	29 (29.5)	31 (50.0)		
Motor fluctuations (*n*, %)	46 (28.8)	20 (20.4)	26 (41.9)	2	0.003
Dyskinesias (*n*, %)	42 (26.3)	18 (18.3)	24 (38.7)	2	0.004
Hoehn and Yahr	2.6 (1.0)	2.1(0,8)	3.2 (1.0)	1	<0.001
Hoehn and Yahr (*n*, %)	(I) 21 (13.1) (II) 63 (39.4) (III) 44 (27.5) (IV) 23 (14.4) (V) 9 (5.6)	(I) 18 (18.4) (II) 52 (53.1) (III) 21 (21,4) (IV) 5 (5.1) (V) 2 (2.0)	(I) 3 (4.8) (II) 11 (17.7) (III) 23 (37.1) (IV) 18 (29.1) (V) 7 (11.3)		
UPDRS III	28.8 (15.6)	23.5 (13.0)	37.2 (15.8)	1	<0.001
FOG (*n*, %)	70 (43.8)	30 (30.6)	40 (64.5)	2	<0.001
FOG questionnaire	4.5 (5.8)	2.7 (4.6)	7.4 (6.4)	1	<0.001
Activities of daily living	73.7 (25.3)	82.1 (19.0)	60.4 (28.3)	1	<0.001
MMSE	26.8 (4.7)	27.6 (3.8)	25.7 (5.8)	3	0.038
Hypertension (*n*, %)	62 (38,8)	38 (38.7)	24 (38.7)	2	0.993
Diabetes (*n*, %)	16 (10.0)	7 (7.1)	9 (14.4)	2	0.130
Stroke (*n*, %)	6 (3.7)	2 (1.9)	4 (6.0)	2	0.208
Neuroimaging of CVD (*n*, %)	35 (21.9)	22 (22.4)	13 (20.9)	2	0.825
Symptomatic orthostasis (*n*, %)	58 (36.2)	33 (33.6)	25 (24.8)	2	0.416
Syncope (*n*, %)	1 (0,6)	0	1 (1.6)	2	0.765

*Abbreviations*. PD: Parkinson disease; UPDRS: Unified Parkinson's Disease Rating Scale; FOG: freezing of gait; MMSE: minimental State Examination; TDT: tremor-dominant subtype; ART: akinetic-rigid subtype; CVD: cerebrovascular disease.

^a^Data are mean (SD), absolute numbers, and percentages.

^b^Test 1 = Mann-Whitney *U* test; test 2 = *χ* ^2^test; test 3 = independent samples *t*-test.

**Table 2:** Functional tests, gait parameters, and drug treatments ^a^.

	All patients (*n* = 160)	Nonfallers (*n* = 98)	Fallers (*n* = 62)	Test^b^	*P* value
Tinetti					
Balance	10.9 (5.7)	13.4 (3.9)	7.1 (6.0)	1	<0.001
Gait	8.6 (4,6)	10.3 (3.2)	5.8 (5.2)	1	<0.001
Total	19.5 (10.2)	23.7 (7.0)	12.9 (11.0)	1	<0.001
Timed up and go (s)	11.4 (8.0)	10.7 (7.9)	13.1 (8.1)	1	0.004
Velocity (m/s)	1.17 (0.31)	1.19 (0.30)	1.10 (0.32)	1	0.293
Step length (m)	0.77 (0.20)	0.79 (0,24)	0.69 (0.22)	1	0.093
Cadence (steps/s)	1.55 (0.70)	1.52 (0.23)	1.61 (0.41)	1	0.253
Levodopa use (*n*, %)	139 (86.8)	84 (85.7)	55 (88.7)	2	0.639
Levodopa dose (mg/d)	557.8 (254.1)	504.1 (208.3)	638.7 (295.8)	1	0.005
Dopamine agonist use (*n*, %)	98 (61.2)	58 (59.2)	40 (64.5)	2	0.511
MAOB-I use (*n*, %)	49 (30.6)	29 (29.5)	20 (32.2)	2	0.728
COMT-I use (*n*, %)	25 (15.6)	10 (10.2)	15 (24.2)	2	0.025
Amantadine use (*n*, %)	13 (8,1)	5 (5.1)	8 (12.9)	2	0.134
Anticholinergic use (*n*, %)	2 (1.2)	2 (2.0)	0		
Polytherapy (*n*, %)	83 (51.8)	49 (50.0)	34 (54.8)	2	0.649
Benzodiazepine use (*n*, %)	22 (13.7)	12 (12.2)	10 (16.1)	2	0.490
Antidepressant use (*n*, %)	18 (11.2)	11 (11.2)	7 (11.3)	2	1.000
Neuroleptic use (*n*, %)	8 (5.0)	1 (1.0)	7 (11.3)	2	0.006
Cholinesterase inhibitor use (*n*, %)	7 (4.3)	0	7 (11.3)	2	0.001

*Abbreviations*. MAOBI: inhibitor of monoamine oxidase B; COMTI: inhibitor of catechol-O-methyl transferase.

^a^Data are mean(SD), absolute numbers, or percentage.

^b^Test 1 = independent samples *t*-test; test 2 = *χ* ^2^ test.

**Table 3:** Logistic regression analysis.

	Bivariate analysis			Multivariate analysis		
	Odds ratio	95% CI	*P* value	Odds ratio	95% CI	*P* value
Age	1.04	0.99–1.08	0.025	1.04	0.95–1.14	0.381
Disease duration	1.12	1.05–1.19	<0.001	0.96	0.89–1.03	0.326
PD subtype	2.37	1.22–4.60	0.010	2.07	0.89–4.80	0.090
Motor fluctuations	2.81	1.39–5.69	0.004	1.91	0.75–5.62	0.200
Dyskinesia	2.80	1.36–5.78	0.005	0.98	0.33–2.87	0.972
Hoehn and Yahr	3.06	2.04–4.60	<0.001	1.59	0.72–3.50	0.247
UPDRS	1.06	1.03–1.09	<0.001	0.98	0.94–1.04	0.658
FOG	4.42	2.24–8.72	<0.001	1.65	0.67–4.03	0.274
FOG questionnaire	1.15	1.08–1.22	<0.001	1.01	0.89–1.22	0.131
Activities of daily living*	0.96	0.94–0.97	<0.001	1.02	0.92–1.14	0.807
MMSE*	0.91	0.85–0.98	0.017	1.02	0.93–1.13	0.640
Tinetti Balance*	0.80	0.74–0.86	<0.001	0.84	0.74–0.97	0.017
Tinetti Gait*	0.80	0.73–0.87	<0.001	1.13	0.95–1.43	0.256
Tinetti total*	0.89	0.85–0.93	<0.001	0.93	0.87–1.01	0.690
Get-up-and-go	0.95	0.90–1.01	0.189			
Levodopa dose	1.002	1.001–1.004	0.005	1.02	0.94–1.19	0.944
COMT-I use	2.80	1.17–6.74	0.021	3.41	1.06–11.37	0.052
Neuroleptic use	12.34	1.48–102.98	0.020	1.25	0.16–12.67	0.854
Cholinesterase inhibitor use	11.92	2.12–12.34	0.002	1.11	0.23–11.78	0.756

*Abbreviations*. PD: Parkinson disease; UPDRS: Unified Parkinson's Disease Rating Scale; FOG: freezing of gait; MMSE: Minimental State Examination.

* Functional tests: higher scores means normality.

**Table 4:** Receiver operating characteristic analyses.

	AUC	Sensitivity	Specificity	Accuracy %	Cut-off value
Tinetti Balance	0.81	0.71	0.79	0.76	11.5
Tinetti total	0.81	0.60	0.86	0.52	17.5
Hoehn and Yahr	0.78	0.77	0.71	0.74	2.5
Tinetti Gait	0.77	0.71	0.74	0.76	10.5
UPDRS III	0.76	0.77	0.70	0.72	26.5
Activities of daily living	0.74	0.73	0.66	0.69	85
Disease duration	0.70	0.71	0.70	0.70	7.5
FOG questionnaire	0.70	0.55	0.84	0.74	8.5
Levodopa dose	0.66	0.79	0.49	0.61	425
Age	0.62	0.52	0.69	0.54	76.5
MMSE	0.60	0.23	0.89	0.63	22.5

*Abbreviations*. AUC: area under the curve; UPDRS: Unified Parkinson's Disease Rating Scale; FOG: freezing of Gait; MMSE: Minimental State Examination.

## APPENDIX

### Tinetti Functional Test [36]

Balance Tests: Subject is seated in hard, armless chair. The following maneuvers are tested.

- Sitting balance
  - Leans or slides in chair  (0)
  - Steady, safe  (1)
- Arises
  - Unable without help  (0)
  - Able, uses arms to help  (1)
  - Able, without using arms  (2)
- Attempts to arise
  - Unable without help  (0)
  - Able, requires >1 attempt  (1)
  - Able to rise, 1 attempt  (2)
- Immediate standing balance  (first 5 seconds)
  - Unsteady  (swaggers, moves feet, trunk sway) (0)
  - Steady but uses walker or other support  (1)
  - Steady without walker or other support  (2)
- Standing balance
  - Unsteady  (0)
  - Steady but wide stance  (heels >10.16 cm [4 inc] apart and uses cane or other support  (1)
  - Narrow stance without support  (2)
- Nudged  (subject with feet as close together as possible, examiner pushes lightly on subject's sternum 3 times)
  - Begins to fall  (0)
  - Staggers, grabs, catches self  (1)
  - Steady  (2)
- Eyes closed  (subject with feet as close together as possible)
  - Unsteady  (0)
  - Steady  (1)
- Turning 360°
  - Discontinuous steps  (0)
  - Continuous  (1)
  - Unsteady  (grabs, staggers) (2)
  - Steady  (3)
- Sitting down
  - Unsafe  (misjudged distance, falls into chair) (0)
  - Uses arms or not a smooth motion  (1)
  - Safe, smooth motion  (2)

Gait Tests: Subject stands with examiner, walks down hallway or across room, first at “usual pace”, then back at “rapid, but safe pace” (using usual walking aids).

- Initiation of gait
  - Any hesitancy or multiple attempts to start  (0)
  - No hesitancy  (1)
- Step length and height
  - Right swing foot does not pass right stance foot with step  (0)
  - Passes left stance foot  (1)
  - Left swing foot does not pass right stance foot with step  (0)
  - Passes right stance foot  (1)
  - Right foot does not clear floor completely with step  (0)
  - Right floor completely clears floor  (1)
  - Left foot does not clear floor completely with step  (0)
  - Left floor completely clears floor  (1)
- Step symmetry
  - Right and left step lengths not equal  (estimate) (0)
  - Right and left steps appear equal  (1)
- Step continuity
  - Stopping or discontinuing between steps  (0)
  - Steps appear continuous  (1)
- Path (estimated in relation to floor tiles, 30.48 cm [12 in] diameter; observed excursion of 1 foot over about 3 m [10 ft] of the course)
  - Marked deviation  (0)
  - Mild/moderate deviation or uses walking aid  (1)
  - Straight without walking aid  (2)
- Trunk
  - Marked sway or uses walking aid  (0)
  - No sway but flexion of knees or back or spread arms  (1)
  - No sway, no flexion, no use of arms, and no use of walking aid  (2)
- Walking stance
  - Heels apart  (0)
  - Heels almost touching while walking  (1)

Balance score …/16

Gait score …/12

Balance score + gait score …/28

Modified from: Tinetti ME. Performed-oriented assessment of mobility problems in elderly patients. Journal of the American Geriatrics Society 1986; 34 : 119–126.
